# Draft Genome Sequence of *Bacillus cereus* Strain F, Isolated from Ancient Permafrost

**DOI:** 10.1128/genomeA.00561-13

**Published:** 2013-08-01

**Authors:** Evgeniy V. Brenner, Anatoli V. Brouchkov, Alexander M. Kurilshikov, Gennady I. Griva, Elena Kashuba, Vladimir I. Kashuba, O. Melefors, Vladimir E. Repin, Vladimir P. Melnikov, Valentin V. Vlassov

**Affiliations:** Institute of Chemical Biology and Fundamental Medicine, SB RAS, Novosibirsk, Russiaa; Moscow State University, Moscow, Russiab; Institute of the Earth Cryosphere SB RAS, Tyumen, Russiac; Department of Microbiology, Tumor and Cell Biology, Karolinska Institutet, Stockholm, Swedend

## Abstract

*Bacillus cereus* strain F was isolated and cultured from a sample of permafrost, aged presumably about 3 million years, on the Mammoth Mountain (62°56′N, 133°59′E). These genome data provide the basis to investigate *Bacillus cereus* F, identified as a long-term survivor of the extremely cold and close environment.

## GENOME ANNOUNCEMENT

Permafrost covers nearly 65% of Russia’s territory and contains microorganisms which have remained viable over millions of years. Although DNA sequences from 10^6^ years have been reported (1, 2), the results are still limited. The preservation of DNA has great significance in studies of evolution, and the extremely cold environments are also being studied as analogues of extraterrestrial habitats. A viable strain “F” of *Bacillus cereus* was isolated from a sample of permafrost aged presumably about 3 million years on the Mammoth Mountain, Central Yakutia, located on the left bank of the Aldan river (62°56′N, 133°59′E), 325 km upstream from the mouth of the River Lena (3). This strain is being cultivated on Luria broth (LB) medium under standard conditions. Although *Bacillus cereus* is capable of forming spores, it still seems astonishing that bacteria trapped in frozen soil can survive soil radiation and other damaging agents at temperatures of nearly –3°C and under the conditions of a closed environment and almost total deprivation of energy sources. Due to its remarkable survival capabilities, *Bacillus cereus* F can be considered a potential model organism for the study of low-temperature adaptations. 

Here we report the first draft sequence of the *Bacillus cereus* F genome. The sequencing was performed with the use of a hybrid approach. At the first stage, a rapid fragment library was sequenced on a Roche FLX instrument following the titanium protocol, resulting in approximately 170 Mb of raw data with a mean read length of 340 nucleotides. A 2 × 50-bp mate-paired library with a mean insertion size of 1 kb was additionally sequenced by using ABI SOLiD v.3.5 Workflow. The combination of different sequencing platforms and library types made it possible to perform *de novo* assembly from Roche FLX reads, followed by scaffolding by SOLiD reads pairing. 

The draft genome sequence includes nearly 5.26 Mb of nucleotide sequences and is characterized by 186 contigs. The genome obtained was annotated using the RAST annotation server. As many as 5,602 coding sequences were predicted. 

Believed to be as ancient as the permafrost sample from which this strain was isolated, *Bacillus cereus* F exhibits a surprisingly high level of homology with modern *Bacillus cereus* strains, particularly with *Bacillus cereus* strain ATCC 10987. The difference in chromosomal nucleotide sequences between these two strains does not exceed 1.5%, which is comparable to or even less than the difference between available chromosomal sequences of other *Bacillus cereus* strains. These observations may reflect the adaptability of *Bacillus cereus* for long-term survival and the evolution strategies of this organism in the permafrost environment (4–6).

### Nucleotide sequence accession numbers. 

This whole-genome shotgun project has been deposited at DDBJ/EMBL/GenBank under the accession number AHHI00000000. The version described here is the first version, AHHI01000000.
